# Intrinsic 40Hz-phase asymmetries predict tACS effects during conscious auditory perception

**DOI:** 10.1371/journal.pone.0213996

**Published:** 2019-04-03

**Authors:** Jan Meier, Guido Nolte, Till R. Schneider, Andreas K. Engel, Gregor Leicht, Christoph Mulert

**Affiliations:** 1 Department of Psychiatry and Psychotherapy, University Medical Center Hamburg-Eppendorf, Hamburg, Germany; 2 Department of Neurophysiology and Pathophysiology, University Medical Center Hamburg-Eppendorf, Hamburg, Germany; 3 Centre for Psychiatry and Psychotherapy, Justus-Liebig-University Giessen, Giessen, Germany; University of Graz, AUSTRIA

## Abstract

Synchronized oscillatory gamma-band activity (30-100Hz) has been suggested to constitute a key mechanism to dynamically orchestrate sensory information integration across multiple spatio-temporal scales. We here tested whether interhemispheric functional connectivity and ensuing auditory perception can selectively be modulated by high-density transcranial alternating current stimulation (HD-tACS). For this purpose, we applied multi-site HD-tACS at 40Hz bilaterally with a phase lag of 180° and recorded a 64-channel EEG to study the oscillatory phase dynamics at the source-space level during a dichotic listening (DL) task in twenty-six healthy participants. In this study, we revealed an oscillatory phase signature at 40Hz which reflects different temporal profiles of the phase asymmetries during left and right ear percept. Here we report that 180°-tACS did not affect the right ear advantage during DL at group level. However, a follow-up analysis revealed that the intrinsic phase asymmetries during sham-tACS determined the directionality of the behavioral modulations: While a shift to left ear percept was associated with augmented interhemispheric asymmetry (closer to 180°), a shift to right ear processing was elicited in subjects with lower asymmetry (closer to 0°). Crucially, the modulation of the interhemispheric network dynamics depended on the deviation of the tACS-induced phase-lag from the intrinsic phase asymmetry. Our characterization of the oscillatory network trends is giving rise to the importance of phase-specific gamma-band coupling during ambiguous auditory perception, and emphasizes the necessity to address the inter-individual variability of phase asymmetries in future studies by tailored stimulation protocols.

## Introduction

**Fig 1:**
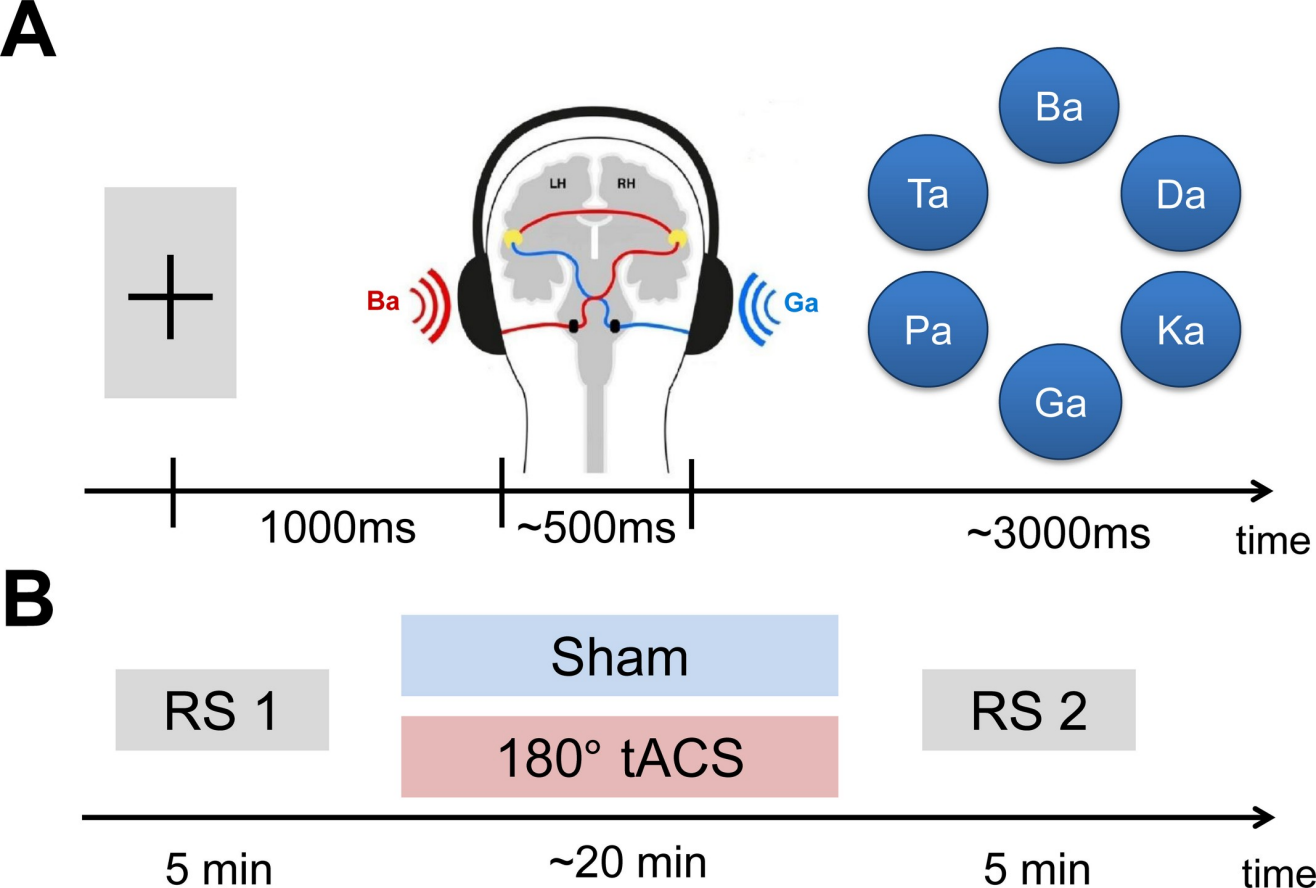
Dichotic listening task and procedure. (A) Exemplary single trial. After 1sec of central fixation, two syllables were presented simultaneously to both ears. After a delay, participants chose the syllable that they perceived out of six alternatives. (B) Procedure. Every subject participated in two sessions (sham and anti-phase tACS) on two different days. The order of sessions was randomized across participants. Every session started with a resting state (RS) EEG, followed by either sham or anti phase stimulation at 40Hz and another resting state EEG.

Synchronized neuronal activity across widely distributed cortical regions is encoded in unique spectral signatures and thought to reflect a key mechanism for cortical information integration and conscious perception in humans [1]. In particular, synchronization in the gamma-frequency range (30–100 Hz) has been associated with feature integration from distant cortical sites [2] and might efficiently route cortical information flow to task-relevant cortical regions [3]. While most of the previous work was done in the visual domain [1–3], recent findings indicated that a similar mechanism might underlie conscious auditory perception [4,5], where information from both ears is integrated across both auditory cortices during a dichotic listening (DL) task (Fig 1A) [6]. In this paradigm, healthy participants typically exhibit the well-known right ear advantage during DL; they report more often the syllable presented to the right than to the left ear [7], which is best explained by the supremacy of contralateral pathways from the speech-dominant left hemisphere [8]. Furthermore, left ear percept is associated with increased functional [4] and effective [5] gamma-band connectivity, which might be mediated by cortico-cortical callosal fibers [6].

Even though most of this evidence is correlative in nature, causal links between oscillatory key signatures during auditory processing and structural connections could be investigated with novel non-invasive brain stimulation techniques such as transcranial alternating current stimulation (tACS), which enable frequency-specific modulation of cortical oscillations [9]. In the past, tACS has been suggested to entrain cortical oscillations in a frequency-specific manner [10–15] and phase-dependent effects have been demonstrated in human [16–22] and animal studies [10,12], making it an ideal tool to probe the causal influence of phase relationships on conscious auditory perception [23,24]. Importantly, highly selective stimulation at different cortical sites can now be implemented by optimized stimulation protocols derived from computational models [14,18,19].

**Fig 2:**
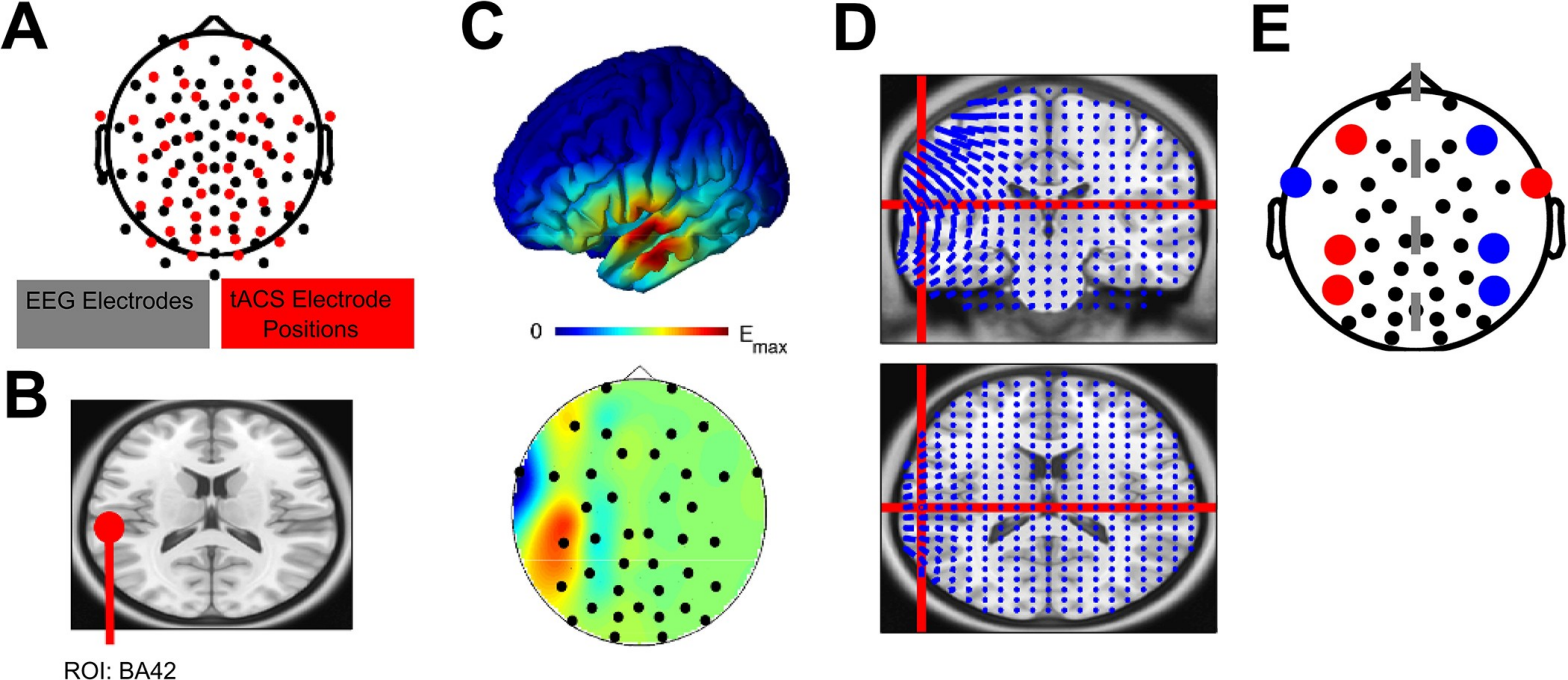
Spatiotemporally-matched tACS. (A) Electrode layout. Black dots indicate the 64 EEG electrodes, while all the red dots indicate the potential positions for tACS electrode placement. (B) Targeted region of interest: area 42. (C) Result of the electric field simulation to target the left BA42. Upper: Resulting electric field on an MNI brain. Lower: 2D topography that highlights which positions should be utilized for stimulation electrode placement. Here, we constrained the electrode placement to the 4 electrodes with the highest contribution. (D) Directionality of the electric field. Note, we modeled the electric field in a way that the field lines were parallel to the assumed tangential dipole orientation in BA42. (E) Resulting asymmetric tACS electrode placement relative to other potential tACS electrode positions (black). Red and blue dots indicate opposite polarities.

In this study, we tested whether the interhemispheric information flow during a dichotic listening (Fig 1A) can be modulated by spatially-matched multi-site 40Hz with a phase-lag of 180° between the left and right auditory cortex (BA42). Since it has been shown that the interhemispheric integration of alternating visual tokens into coherent motion percept can reliably be inhibited by 40Hz-tACS with a phase-lag of 180° between hemispheres [18,25], it is conceivable that interhemispheric auditory processing could be selectively altered using a similar stimulation protocol with a tailored high density (HD)-electrode montage derived from current flow modeling (Fig 2). We thus hypothesized that 40Hz-tACS with a phase-lag of 180° between hemispheres should inhibit network synchrony and thereby increase the laterality index.

Whilst previous studies support the concept that inter-areal gamma-band synchronization entails a delayed non-zero phase relationship [4,5,26], the associated metrics (lagged phase synchronization [27], isolated effective coherence [28]) however do not permit the deduction of a specific phase asymmetry between the left and right auditory cortices in degree notation.

To address this issue, we employed an exploratory control analysis to establish a link between the behavioral outcome of the anti-phase stimulation and the individual phase asymmetry during the sham session, recorded with 64-channel electroencephalography (EEG). Hence we investigated whether the time courses of the intrinsic phase asymmetry at 40Hz differed between left and right ear percept, and specifically assessed the circular-linear correlation between the intrinsic phase asymmetry and the behavioral tACS-related modulation.

## Materials and methods

### Participants

Twenty-nine healthy participants were recruited from the University Medical Center in Hamburg, Germany. All subjects were right-handed according to the Edinburgh handedness-scale [29], reported no history of neurological or psychiatric disease, filled out a sociodemographic questionnaire and further provided written informed consent and were paid for participation. Please note that we assessed sociodemographic data as a standard procedure to allow for potential comparisons of healthy control samples with clinical samples. Since no association between sociodemographic factors and early auditory perception in healthy participants had previously been found, we did not further report these data in this manuscript.

Normal hearing was validated by pure tone audiometry for frequencies between 125 and 8000Hz (Esser Home Audiometer 2.0). No participant exhibited interaural differences larger than 9 dB or an auditory threshold above 25 dB. The study was approved by the ethical committee of Medical Association Hamburg (Reference Number: PV4911) and conducted in accordance with the Declaration of Helsinki. One subject with insufficient data quality and two subjects with excessive error rates in task performance (>2 SDs over the mean in a session) were excluded. The remaining 26 subjects (18 men, range: 18–49 yrs, *M* = 28.5 yrs, *SD* = 7.9 yrs) were included in the final analysis.

### Stimuli and procedure

We utilized the Bergen dichotic listening task [4,30], where six consonant-vocal (CV) syllables were coupled and presented simultaneously to each ear via closed headphones (Sennheiser, HAD 200) at 75 dB. We ruled out effects of syllable voicing by combining only syllables with the same voice onset time, which yielded 12 dichotic CV pairs. Voice onset time is characterized by the length of time between the release of a consonant and the onset of voicing. Three syllables (/ba/, /da/, /ga/) were voiced and had a short voice onset time (17-32ms), the other three syllables (/pa/, /ta/, /ka/) were unvoiced with a long voice onset time (75–80 ms). Stimulus onset was temporally aligned and lasted for 400-500ms.

All subjects took part in both (single-blinded) tACS sessions on two different days (*M* = 2.65 days; range: 1–12 days), while the session order was counterbalanced (Fig 1B). After performing the hearing test and filling out all questionnaires, the participants performed 6 practice trials on the day of the first session to get familiarized with experimental procedure and stimulus material. 240 trials were randomly presented in 2 blocks during each tACS session (sham and anti-phase). Every trial started with the participant fixating a central fixation cross for one second, then a syllable combination was played through the headphones and participants indicated their choice from a circular formation showing all six syllables. Participants navigated through the alternatives by left mouse button clicks and confirmed their choice with a right button click. A fixed inter-stimulus interval of 1s was applied between the offset of the visual presentation and the ensuing auditory stimulus. Hence, the trial duration varied between 3.5 and 6.5s in dependence of the individual reaction time. After a fixed delay of 1s, the next trial started. The participants were instructed to report the syllable that they understood most clearly between all 6 syllables, while they were not informed that each trial consisted of two different syllables. Furthermore, we encouraged them to fixate on the cross, relax, reduce head and eye movement and avoid jaw muscle contraction.

We ran the experiment in an electrically shielded and soundproof cabin, where participants were seated with a distance of 60cm in front of a BenQ XL2420T screen (1920 x 1080, 120 Hz). Stimulus presentation was controlled via Presentation software (Neurobehavioral Systems, Albany, CA).

### EEG acquisition and tACS

EEG and tACS Ag/AgCl electrodes were mounted in a custom-made elastic cap for 104 electrodes (Easycap). EEG recordings were obtained from 64 Ag/AgCl electrodes (no amplitude clipping, impedances <15 kΩ, referenced to FCz) using a slightly abrasive electrolyte gel (Abralyt 2000, Easycap). EEG was recorded during all conditions (Resting State 1, Sham, Verum, Resting State 2) using BrainAmp amplifiers (Brain Products GmbH). Signals were sampled at 5 kHz, amplified in the range of ±16.384 mV at a resolution of 0.5 μV and stored for offline analyses.

Transcranial stimulation was applied via a battery-operated stimulator (DC-Stimulator Plus, NeuroConn) using eight Ag/AgCl electrodes (12 mm diameter, Easycap). Electrode placement was based on a current flow model, which was optimized to target the auditory cortex based on 40 available electrode positions (Fig 2A). The combined impedance of all electrodes was kept below 5 kΩ, as measured by the NeuroConn stimulator, using Signa electrolyte gel (Parker Laboratories Inc.). A sinusoidally alternating current of 1,000 μA (peak-to-peak) was applied at 40Hz continuously for 20 minutes during each session. During sham and real stimulation the current was ramped up over 10 seconds to 1,000 μA, but discontinued during the sham condition. All subjects confirmed that stimulation was acceptable and mainly noticeable during the ramp-in phase. It did not induce painful skin sensations or phosphenes. On debriefing, 50% of the subjects were able to correctly guess which tACS-session was assigned to T1 and T2, which confirmed that single blinding was successful.

### Data analyses

The data were analysed in Matlab R2017a using the EEGlab [31] and CircStat [32] toolboxes, custom-written scripts, and the LORETA KEY software package [33] (The KEY Institute for Brain-Mind Research, Dept. of Psychiatry, University Hospital Zurich, Switzerland, http://www.uzh.ch/keyinst/loreta.htm).

#### Behavioral data

We assessed the distribution between right ear and left ear reports by means of a laterality index (LI), ranging from -100 to 100 according to the following formula:

$$LI=\frac{100*(correct\;RE\;reports-correct\;LE\;reports)}{\left(correct\;RE\;reports+correct\;LE\;reports\right)}$$ (1)

while behavioral modulation was computed as

$$LI{}_{mod}=LI{}_{Verum}-LI{}_{Sham}$$ (2)

As a result, positive LI-values indicate a bias towards right ear reports; negative LI-values indicate more left ear reports and a value of zero signals a perfectly balanced distribution between left and right ear reports.

#### EEG data preprocessing

Since no artifact removal approach that reliably reconstructs EEG phase properties is known so far [34,35], we focused all EEG analyses on the sham session.

First, we removed noisy channels, downsampled the data to 250Hz and filtered the signal in the range from 1–100 Hz using two-pass finite element impulse response (FIR) filters as implemented in EEGLab. Moreover, we filtered out line noise at 50 Hz and its harmonics. The filtered data were visually inspected using the raw signal as well as a Fast Fourier transformation (FFT) to ensure that all artifacts were successfully suppressed. Then, removed channels were interpolated by spherical spline interpolation. Epochs containing saccades, noise or excessive muscle artifacts were removed after visual inspection, and all channels were re-referenced to a common average. Subsequently, an independent component analysis (ICA) was employed to identify blinks, eye movements, electrocardiographic and saccadic spike potential artifacts with regard to time courses, characteristic topographies and frequency distributions [36,37]. Finally, DL-data were segmented into 400ms-epochs, starting 200ms before stimulus onset (Fs = 250Hz, 100 time points), and separated by perceptual outcome (left or right ear percept). Out of 240 trials, an average of *M*±*SD* = 76.15±22.02 left ear trials (min: 38; max: 129) and *M*±*SD* = 119.85±20.18 right ear trials (min: 93; max: 155) remained for the analysis of the EEG phase signature.

Importantly, the sample size bias affects the comparison of averaged electrophysiological measures in sensor space [38,39], and even more heavily in source space analyses due to its additional influence on the applied spatial filters [40]. Since matching the trial numbers across conditions within each subject would not sufficiently control for a sample size bias with respect to the ensuing circular-linear correlation analysis, we decided to rule out confounding influences of unequal trial numbers on the individual phase asymmetries by randomizing across conditions *and* subjects.

Hence, we randomly subsampled 38 trials (lowest number across all subjects) out of each subject's datapool for the left and right ear condition, respectively: In this procedure, all trials of each participant were stored in a Matlab-array, which was subsequently randomly permuted using the function *shuffle*.*m*. The first 38 trials along each permuted trial dimension were selected for both ear conditions separately. All instances of the presented data analysis relate to the first randomly selected sample of trials. In total, an absolute number of 3120 trials was discarded throughout the subsampling procedure. Crucially, we repeated this subsampling procedure in a supplementary analysis to confirm that our results were not restricted to one trial selection (see S2 Text, S2 Fig and S2 Table).

#### Source space analyses

Next, the preprocessed data were projected into source space using the LORETA KEY software. We calculated a transformation matrix for all 60 electrodes using exact LORETA zero-error tomography. Based on previous findings [4,5], we decided to focus on the secondary auditory cortex (BA42) given its functional relevance in early auditory perception and syllable perception in particular [41,42]. The ROIs were defined according to the Talairach-Atlas [43] as implemented in the LORETA KEY software. Importantly, we exploited the tangential dipole activity (z-component of the current density vector) in the centroid voxel of BA42 because this dipole component corresponds best to the time window of interest (-200 to 200ms), hence to its underlying neural generators covering the Planum temporale [44–46]. Having extracted the tangential auditory dipole activity and at 40Hz, we computed the asymmetry Δφ for each time point *t* by deriving the angle *φ* of the complex conjugate product of the Hilbert-transformed data with the following formula:

$$\Delta \varphi \;(t)=|\varphi \left(hilbert(x{}_{left}(t))\times conjugate(x{}_{right}(t))\right)|$$ (3)

where

0≤Δφ(t)≤π (4)

and

$$-\pi \leq \varphi \left(hilbert(x{}_{left}(t))\times conjugate(x{}_{right}(t))\right)\leq \pi$$ (5)

Finally, we calculated each participant's average time course of Δφ across trials for each time point (*circ_mean*.*m* function).

#### Statistical analyses

Unless stated otherwise, the significance level was set to α = .05 in all tests, and all mean values are reported with standard deviation values (*M* ± SD). All circular data were processed using the CircStat toolbox. Correlations between behaviour and phase dynamics were assessed as:

$$\rho =\sqrt{\frac{r{}_{xs}^{2}+r{}_{xc}^{2}-2\times r{}_{xs}\times r{}_{xc}\times r{}_{cs}}{1-r{}_{cs}^{2}}}$$ (6)

where r_xc_, r_cs_ and r_xs_ are defined as

$$r{}_{xc}=corr(x,\sin\left(\varphi \right))$$ (7)

$$r{}_{xs}=corr(x,\cos\left(\varphi \right))$$ (8)

$$r{}_{cs}=corr(sin(\varphi ),\cos\left(\varphi \right))$$ (9)

with φ being the circular and x being the linear variable (*circ_corrcl*.*m* function).

In contrast to repeated measures analyses of variance (RM-ANOVA), permutation-based cluster statistics do not depend on assumptions about the data distribution due to their non-parametric nature [47]. Thus, we assessed differences in time courses of the phase asymmetries between left and right ear trials percept trials (100 time points, -200 to 200ms) with a non-parametric permutation test for paired conditions where a permutation distribution was computed by randomly switching the condition labels within participants in each of 10.000 iterations. To address the issue of multiple comparisons, we here report the p-values using the statistics of the maximum difference (maxstat-method, see [47]) after 10.000 permutations.

Since we expected a clear right ear advantage for syllable perception in right-handed individuals, we first conducted two separate t-tests (paired samples, Bonferroni-corrected) to prove that syllables were more often reported through the right ear than through the left ear during both sham- and verum-tACS.

The influence of tACS on the laterality index was assessed with a two-sided t-test for paired samples. Furthermore, the distributions of LI-values during both tACS-sessions were checked for normality with Lilliefors test. Effect sizes were quantified by means of Cohen's d (t-test). We additionally calculated a Bayes factor expressed as *BF10* for the hypothesized effect of tACS on the laterality index with a default scale factor of *r* = 0.707.

## Results

### Behavioral performance during sham- and verum-tACS

The right ear advantage was present during both sham- (LI_Sham_: *M* = 23.714±18.557) and verum-tACS (LI_Verum_: *M* = 24.756±21.535) as participants perceived significantly more syllables presented to the right ear (sham: *M* = 134±19.779; verum: *M* = 136±23.841) than to the left ear (sham: *M* = 83±22.258; verum: *M* = 82±24.661), which was confirmed by two-sided t-tests for both tACS-sessions (sham: *t*(25) = 6.480; *p* < .001, *d* = 2.43; verum: *t*(25) = 5.809; *p* < .001 *d* = 2.22). Moreover, behavioral performance was normally distributed during both sham- (*p* = .50) and verum-tACS (*p* = .50).

Twenty-three out of 26 participants showed a positive LI during both sessions, whereas 3 participants had a negative LI. Across all participants, reporting a syllable that was not presented occurred in 9.311%±5.276% of cases during sham-tACS and in 8.862%±4.489% of cases during verum-tACS.

### Intrinsic 40Hz phase asymmetries predict stimulation outcome

**Fig 3:**
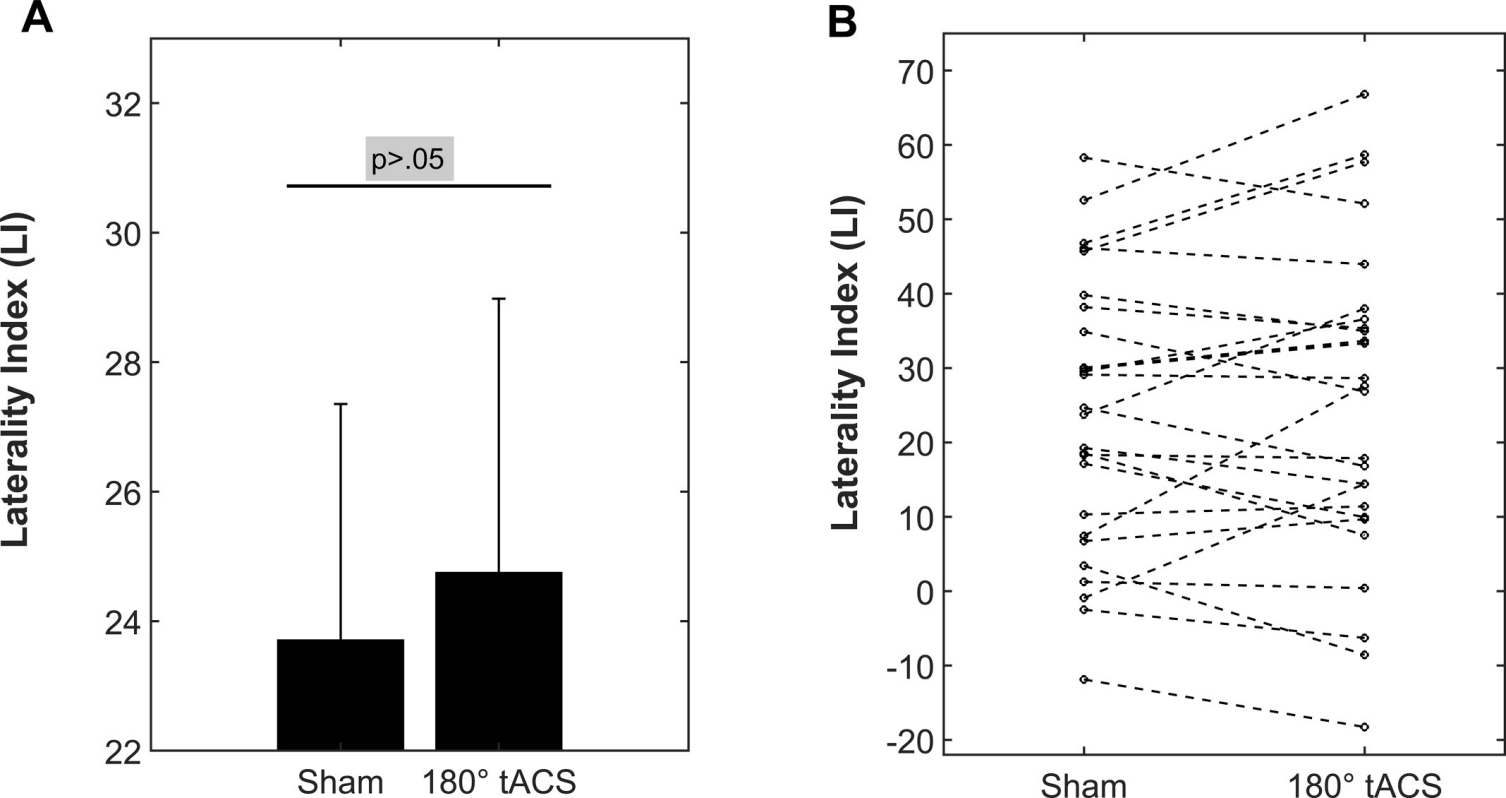
Behavioral results. Laterality Index (LI): Positive values indicate a bias towards right ear reports. (A) 180° tACS at 40Hz does not increase the LI (one-sided t-test for paired-samples, *t*(25) = 0.597, *p* = .278, *d* = 0.05). The error bars depict the standard error of the grand average behavioral performance (LI) during both conditions (mean ± SEM). (B) The individual behavioral performances (*N* = 26) during both conditions. The dashed lines highlight the directionality of the individual modulations (up: increase of LI; down: decrease of LI).

The main influence of tACS on behavioral performance was assessed in a two-sided t-test on the LI values during sham and verum-tACS. This did not confirm the hypothesized increase of the LI (Fig 3A; *t*(25) = 0.597, *p* = .556, *d* = 0.05), which suggests that 40Hz-tACS applied in this electrode montage (Fig 2E) did not consistently amplify the right ear advantage.

The absence of a general tACS effect on behavioral performance, as indicated by a Bayes factor of *bf10* = 0.244, raised the question whether the individual stimulation outcome might depend on the inter-individual differences in oscillatory phase dynamics between the left and right secondary auditory cortices (BA42) at 40Hz. Accordingly, if interhemispheric phase differences predicted a perceptual shift to the left or the right ear, this should be indicated by a circular-linear correlation between the intrinsic phase asymmetries between the left and right BA42 at 40Hz and the difference of LI-values during verum- and sham-tACS. Hence we calculated each participant's phase asymmetry at 40Hz during the sham session by extracting the angle of the complex conjugate product of the Hilbert-transformed source space data.

**Fig 4:**
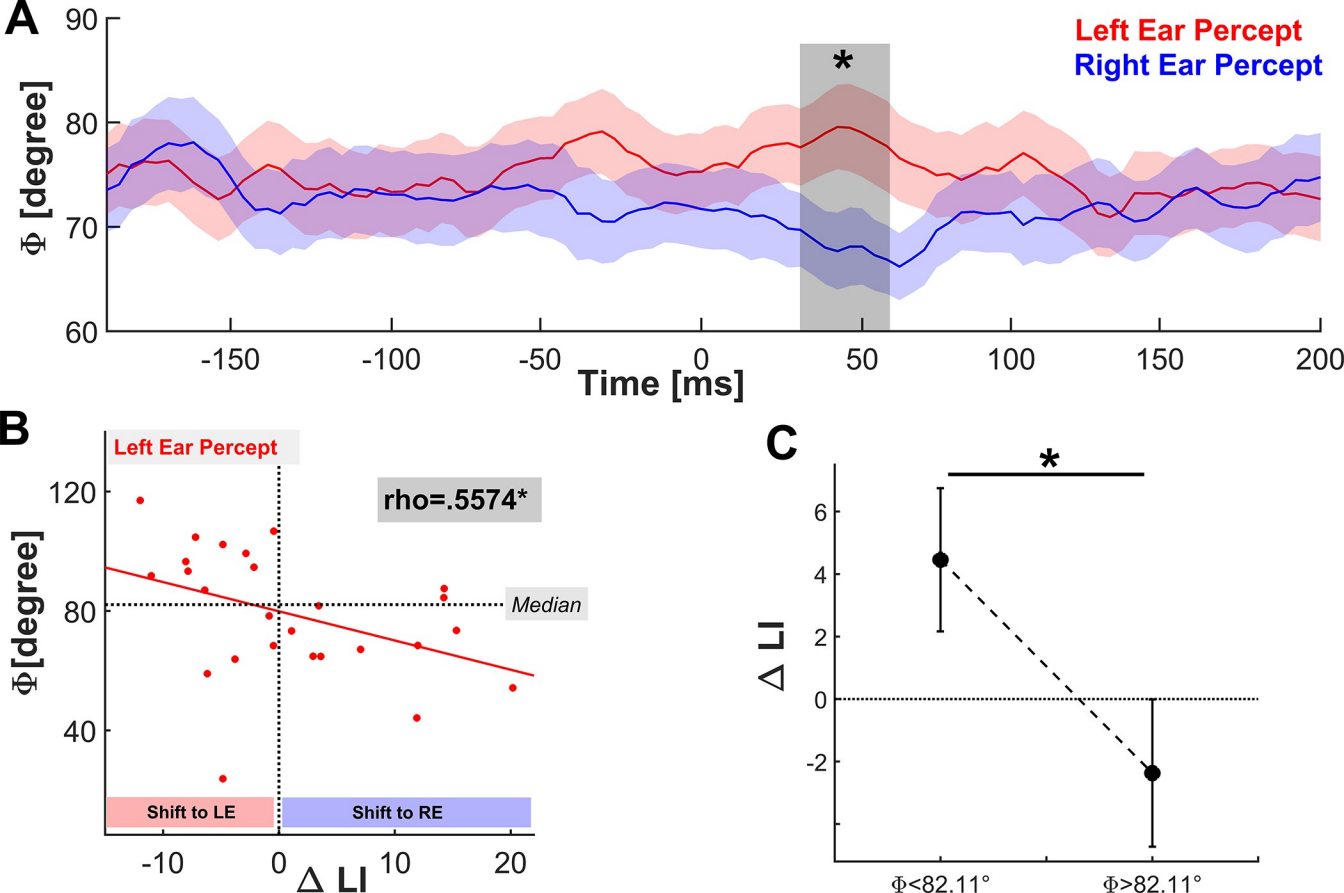
Oscillatory key signature of the interhemispheric phase lag. (A) Time course of the interhemispheric phase difference at 40Hz between the left and right BA42 averaged across all subjects (*M*±*SEM*) during sham-tACS. The shaded bar highlights the interval (36-56ms) where the phase shifts were statistically different between conditions (paired-sample permutation test with 10000 permutations, 'tmax'-method, *p < .05). (B) Circular-linear correlation between the individual phase shifts during auditory processing through the left ear in the cluster-corrected time window (36-56ms) and the behavioral outcome of the 180° stimulation at 40Hz (ΔLI = LI_Verum_—LI_Sham_). The significant correlation (*rho* = .557, *p* = 0.0176) indicates that tACS amplified the right ear advantage in subjects whose oscillatory asymmetry at 40Hz was smaller (closer to 0°) during conscious auditory processing. Contrary, augmented interhemispheric asymmetry (closer to 180°) was associated with a shift to left ear processing. (C) tACS-effect on behavioral performance after splitting the sample at the median angle (φ = 82.11°) into two equally sized subgroups (*N* = 13).

After dividing all trials into left or right ear responses, circular means were calculated across trials for each time point (-200ms to 200ms post-stimulus onset interval) in each subject. We applied a non-parametric paired sample permutation test to investigate whether the across participant phase asymmetry at 40Hz differed between left and right ear percept in a specific time period. The permutation test revealed that the phase asymmetries of the perceptual outcomes differed significantly in the post-stimulus onset interval from 36-56ms (Fig 4A; LE percept: 79.1°±20.8°; RE percept: 67.8°±18.1°; circular mean±SD; Permutation Test 't-max'-Method, multiple comparison corrected *p*-values are displayed in Table 1). Clearly, the grand average phase asymmetry at 40Hz between the left and right BA42 was augmented during left ear percept compared to right ear percept in this time window. As participants with a negative LI might exhibit an atypical organization of speech perception due to an altered interhemispheric communication between auditory cortices [6,48], we repeated the non-parametric permutation test after excluding three participants with an atypical LI to rule out potential confoundations (S2 Text). Importantly, the exclusion of these participants again yielded a significant difference between the perceptual responses of the phase asymmetries in the post-stimulus onset interval from 44-60ms (see S1 Table; Figure A in S1 Fig).

**Table 1:** Corrected *p*-values for permutation statistics. Corrected *p*-values (Tmax-method) for the non-parametric paired sample permutation test (Fig 4A), which was applied to the intrinsic phase asymmetries at 40Hz during left ear and right ear processing. The permutation distribution was computed by randomly switching condition labels within participants in each of 10.000 iterations.

epoch	32-36ms	36-40ms	40-44ms	44-48ms	48-52ms	52-56ms	56-60ms
***p*-value**	.1892	.0455	.0189	.0304	.0477	.0406	.0501

Having identified a specific time window that revealed a significant difference between conditions, we next tested our hypothesis that the individual auditory asymmetries predicted the behavioral modulation by tACS. Therefore, we computed one circular mean across all time points in this post-stimulus onset interval (36-56ms) across each subject's left ear trials during sham-tACS and assessed the circular-linear correlation between these second order means of the intrinsic phase asymmetry and the individual tACS modulations (LI_mod_). Interestingly, a significant correlation (*rho* = .557, *p* = 0.0176) confirmed our assumption that the behavioral modulation depended on the temporal asymmetry: Stronger phase asymmetries (closer to 180°) were associated with a perceptual shift to left ear processing, whereas an amplification of the right ear advantage was associated with weaker phase asymmetries (closer to 0°). This was further supported by a subgroup analysis after performing a median split on phase asymmetries during left ear percept to divide into low (φ<82.11°) and high (φ>82.11°) asymmetry: The tACS-modulation was significantly elevated in subjects with phase asymmetries above 82.11° (LI_mod_: *M* = 4.455±8.257) compared to subjects with asymmetries below 82.11° (LI_mod_: *M* = -2.371±8.485) (Fig 4C; *t(*24) = 2.079; *p* = .049; *d* = 0.815). Please note that the median split analysis was performed for illustration purpose to highlight the bidirectional impact of the stimulation. Furthermore, the significant circular-linear correlation between tACS-related behavioral modulation and the intrinsic phase asymmetry (*rho* = .5932, *p* = 0.0175) during left ear percept in the sham session was not affected by the exclusion of participants with an atypical LI during the sham session (Figure B in S1 Fig).

Since the participants performed the DL task during sham- and verum-tACS on two different days, this dataset could not yield information about the test-retest reliability of the intrinsic phase asymmetry. To determine this, we analyzed pilot data (*N* = 18) from another experiment where the DL task was performed during 64-channel EEG recording on two different days. Crucially, the phase asymmetry values at 40Hz exhibited a high test-retest reliability during left ear processing (*rho* = .8529; *p* = .0047; see S3 Text, S3 Fig).

Collectively, the above findings reveal that high frequency phase asymmetries in the gamma-range exhibit different temporal profiles during ambiguous auditory perception, and that the individuality of these spectral asymmetries predicts the outcome of the electrical stimulation on a behavioral level.

## Discussion

In this study, we tested whether (1) the transcallosal information flow between the left and right SAC can be modulated during conscious auditory perception with high-frequency tACS at 40Hz, and (2) to what extent the stimulation outcome was associated with the individual asymmetries of the spectral profiles.

Clearly, our bilateral HD-montage at a phase-lag of 180° failed to elicit a general effect throughout all subjects. Since the participants responded differently to our fixed stimulation protocol, we performed an exploratory source space analysis to derive an oscillatory key signature of the phase asymmetry at 40Hz during dichotic listening. Our EEG-analysis of the phase dynamics demonstrated that syllable perception through the left ear does not only depend on elevated functional [4] and effective [5] gamma-band coupling, but also that its mean coupling direction at 40Hz differs significantly from right ear processing. At first sight, the finding of increased phase asymmetry during left ear percept may contradict the idea that transcranially decoupling the left and right auditory cortex with a phase-lag of 180° causes a shift to right ear processing. Consequently, elevated interhemispheric coupling and ensuing shift to left ear percept would be expected by a stimulation with zero-lag between hemispheres. In accordance with that, the original communication through coherence hypothesis (CTC,[49]) initially proposed zero-phase synchronization in the gamma-frequency range as the key mechanism for bidirectional coupling between two neuronal groups, whereas phase synchronization in lower frequencies was suggested for enhanced delays in increasingly distant cortico-cortical communication. However, more recent studies evidenced that bidirectional coupling through gamma-band coherence entails directedness with a systematic delay [26,50–52], and thus does not occur at zero phase. Importantly, this was further supported by EEG studies investigating dichotic listening: Increased functional connectivity during left ear percept should reflect a shift away from 0°, because the associated metric (lagged phase synchronization,[27]) suppresses zero phase-lag contributions [53]. In line with that, another study [5] exploiting source space effective connectivity analysis during dichotic listening revealed elevated isolated effective coherence (iCoh,[28]) for left ear percept from the right to the left BA42 compared to the other direction as well as compared to perception through the right ear. Delayed (non-zero) lag inter-areal gamma-band synchronization is visible in Granger-causal influences and iCoh specifically [28], because it signifies that variance in one oscillation explains unexplained variance in another oscillation several milliseconds later. Collectively, our characterization of the intrinsic phase asymmetry supports the above mentioned studies in that long-range auditory synchronization in the gamma-band range enables conscious auditory perception through the subdominant ear with a non-zero phase-lag.

Here, we characterized phase asymmetries as an oscillatory network trend which exhibited considerable inter-individual variation across our sample (range: 24°-117°, see Fig 4B), and argue that the assessment of phase asymmetries might be a crucial network parameter to carefully consider, in order to optimize multi-site stimulation protocols with tailored phase-lags between the targeted oscillators. This is further supported by the fact that the asymmetry values showed a high test-retest reliability (see S3 Text, Figure B in S3 Fig), which suggests that phase asymmetries could indeed reflect a robust auditory network trend that exhibits low intra- and high inter-individual variability in a specific frequency range.

To date, tACS is debated as a highly-promising tool to non-invasively probe the causal influence of neuronal oscillations for a variety of cognitive functions [9,54], while its impact on large-scale networks heavily depends on a broad variety of parameters such as stimulation intensity [55], waveform and envelope [56,57], network state [58,59] or the electrode montage [18,25]. So far, it appeared to be the nature of non-invasive brain stimulation that its effects on physiology and behavior are often small [60], whilst the publication bias further impedes critical discussion on disadvantageous study protocols with regard to crucial stimulation parameters, such as intensity, montage frequency and phase-lag. In this study, our control analysis demonstrated that the behavioral outcome of the 180°-stimulation depended on the phase asymmetry: Elevated phase asymmetry was associated with a shift to left ear processing, while the right ear advantage was amplified when the asymmetry was closer to 0° (Fig 4B). Consequently, the subgroup division at the median angle of 82.11° revealed a bidirectional impact of our stimulation (Fig 4C), suggesting that the asymmetric nature of conscious auditory processing can selectively be modulated by spatiotemporally-matched tACS. Moreover, these findings support the concept that synchronized gamma-band activity not only mediates the integration of visual [18,25,61,62], but also auditory information from both hemispheres [63]. However, the circular-linear relationship raises the question how the external 40Hz driving force interacted with the intrinsic phase relationship of the neuronal oscillators in the left and right secondary auditory cortex. We argue that the selective modulation of conscious auditory perception might depend on the deviation of the exogenous from the endogenous phase lag: The interhemispheric network was prone to inhibition when the intrinsic lag differed strongly from the transcranially-induced 180°-lag, whereas a shift to left ear percept was facilitated when the deviation of the tACS-induced lag from the intrinsic lag was low. Hence, it is conceivable that long-range gamma-band synchronization can be efficiently amplified if the external driving force mimics an electrical field bilaterally with the intrinsic phase asymmetry. Accordingly, the cortical network dynamics should be most efficiently hampered if the deviation of the exogenous phase lag from the intrinsic lag approximates π.

Since schizophrenic patients with auditory-verbal hallucinations (AVH) exhibit increased interhemispheric gamma-band coupling during dichotic listening and thus a reduced right ear advantage [64–66], the current study was initially designed to increase the laterality index, which might offer a potential application of tACS in normalizing disturbed gamma-band connectivity underlying AVH in patients with schizophrenia. Our results suggest that the characterization of the intrinsic phase relationship in the gamma-band range might benefit tailored tACS protocols in future studies.

Importantly, the interindividual variability in shape and size of the targeted pathway was highlighted by a study that utilized Diffusion Tensor Imaging (DTI) of the CC with a focus on posterior subregions connecting the auditory cortices: Stronger anatomical connectivity between these areas was associated with augmented left ear processing [67]. Even though our data do not provide tractographic information about the CC, it is conceivable that the interindividual differences in angular asymmetries at 40Hz might relate to individual variation of structural features of the transcallosal auditory pathways, and that these phase oscillations reflect undulations of neuronal excitability [68]. Such phase-related interindividual differences in the gamma-band level out in the grand average across subjects, which may explain the absence of a general behavioral effect by 180°-tACS across all participants.

Several studies have pointed out the role of slow wave oscillatory dynamics for hearing [19,21], speech perception [24] and syllable perception in particular [69]. Here we provide evidence that high-frequency oscillations in the gamma-band range might not only shape auditory perception in terms of magnitude properties [70], but in terms of the individual interhemispheric phase signature. In our experiment, our effects are better explained by 40Hz-phase properties given that we applied the alternating currents at equal intensities to each hemisphere, while the phase asymmetry interacted with the [53]advanced protocols can selectively modulate long-range cortico-cortical signal transmission with phase-dependent effects in different modalities [16,18–20,71,72].

### Confounds and limitations

A number of limitations hamper the analysis of gamma-band activity and long-range coupling in human EEG recordings, such as the effects of volume conduction in the cortical tissue, broadband muscle activity that might obscure physiologic gamma-band signatures or the low spatial resolution of EEG recordings. We addressed these issues by analyzing all data at the source space level using the eLORETA approach after carefully removing artifacts by means of an ICA [37]. In addition, we employed connectivity analyses which reduce the impact of volume spread and allow to estimate the directionality of these effects [53].

A further potential issue is the statistical validity of the grand average phase asymmetry time courses (Fig 4A), as each averaging and trial subsampling method has some limitations. To control for a sample size bias, we randomly selected a subsample of 38 trials for each participant and thus discarded event-related data from ensuing analyses. Importantly, this method was exploited in another EEG study investigating long-range connectivity estimates in source space [40] to avoid an additional sample size bias to spatial filters; and was further discussed as a valid method to compute grand average images across subjects and conditions [73]. Crucially, matching the trial numbers within subjects would not correct for a sample size bias with respect to circular-linear correlation analyses. Furthermore, our goal was to keep results comparable with our supplementary reliability analysis (see S3 Text, S3 Fig), as classical test theory demands an equal number of observations throughout all subjects for the assessment of reliability scores [38]. In this study, we accepted a minimum number of 38 trials since previous studies had demonstrated that an adequate reliability estimate of 0.8 can be obtained at a minimum number of 21 trials in healthy control groups [74], as well as that averaging across 30 trials can yield sufficient test-retest reliabilities for early event-related potentials [75,76]. Importantly, our supplementary reliability analysis provided evidence that the respective metrics (laterality index, intrinsic phase asymmetry) are robust across days (see S3 Fig, S3 Text), as well as that other random trial selections yielded similar results (see S2 Fig, S2 Table).

To date, several studies have pointed out that the recovery of true oscillatory activity during electrical stimulation is not only hampered by linear, but non-linear components of the complex tACS artifact in particular [34,35,77], indicating that current approaches such as beamforming [78,79] or artifact template subtraction [11] fail to reliably reconstruct the EEG-signal. Since we did not attempt to disentangle the artifact from brain activity, our data—along with other studies employing current artifact removal techniques—do not provide evidence for the exact electrophysiological mechanisms acting *during* stimulation. While several studies suggested entrainment of neuronal oscillations as the key mechanism of the observed tACS effects [10–13,15], other reports interpreted the effects as plastic changes [80,81] or attenuated neuronal adaptation [82].

However, the interhemispheric network communication between the left and right auditory cortices during ambiguous syllable perception has been investigated over decades exploiting multimodal imaging methods [4–8,30,65,67,83], hence establishing a reliable neuronal framework for its behavioral measures. Collectively, we can argue that (1) the laterality index reflects hemispheric specialization for language, while its magnitude is related to inter-individual trait differences in transcallosal topography, mainly the posterior third of the CC connecting the auditory cortices [6,67], (2) the perception of syllables through the left ear is accompanied by elevated functional [4] and effective gamma-band coupling [5] between the left and right BA42, and that (3) oscillatory phase dynamics at 40Hz reveal different time courses between left and right ear percept in terms of interhemispheric asymmetry. Based on this evidence, it is plausible to assume that the external 40Hz driving force interacted with the intrinsic phase relationship of the neuronal oscillators in the left and right secondary auditory cortex. In this context, the employment of tailored HD-tACS protocols might be crucial to modulate cortico-cortical network communication by non-invasive brain stimulation.

### Conclusions

In summary, our results support and expand the idea that interhemispheric gamma-band phase dynamics mediate conscious auditory perception [4,5] and demonstrate the potential of HD-tACS to selectively modulate frequency-specific large-scale cortical networks. However, the parameter space of tACS is not very well explored yet and it is unclear which stimulation parameters should be utilized to maximize its physiological efficacy [60,84]. Importantly, this study provides novel insights into how the intrinsic phase relationship can be exploited as a significant network parameter for the implementation of optimized stimulation protocols.

In the future, it might be possible to tailor therapeutic interventions by means of spatiotemporally-matched multi-site HD-tACS for certain neuropsychiatric diseases such as autism spectrum disorders [85], Parkinson’s disease [86] or schizophrenia [66,87,88] that have previously been associated with impaired network synchronization [85,89]. In particular, auditory hallucinations in schizophrenia have been suggested to reflect an over-coupling between auditory and frontal areas [83,90]. The present findings underline the idea that tACS might be an ideal candidate for potential treatment of network disorders [91,92].

## Supporting information

S1 Text Electric field modeling. (DOCX)

S2 Text Supplementary control analyses. (DOCX)

S3 Text Test-retest reliability of the laterality index and the intrinsic phase asymmetry at 40Hz. (DOCX)

S1 Fig Oscillatory key signature of the interhemispheric phase lag after excluding participants with an atypical laterality index (*n* = 23). (JPG)

S2 Fig Sensitivity analysis of the 40Hz intrinsic phase asymmetry. Average time courses (solid lines) of the intrinsic phase asymmetry (related to Fig 4A in the manuscript) with standard errors of the circular means (dashed lines) for 10 different randomized trial selections (*M ± SE* over 38 trials during left (red lines) and right (black lines) ear percept, respectively). The turquoise-shaded bar highlights that the effect of increased phase asymmetry during left ear percept was present in the marked post-stimulus onset interval throughout all repetitions (*maxstat*-method; corrected *p*-values are displayed in S2 Table).
(JPG)

S3 Fig Test-retest reliability of the laterality index and the intrinsic phase asymmetry at 40Hz during left ear percept. (JPG)

S1 Table Corrected *p*-values for supplementary permutation statistics (related to S1 Fig). Corrected *p*-values (*tmax*-method) for the non-parametric paired sample permutation test (related to Figure A in S1 Fig), which was applied to the intrinsic phase asymmetries at 40Hz during left ear and right ear processing after excluding three participants with a negative LI (*n* = 23). The permutation distribution was computed by randomly switching condition labels within participants in each of 10.000 iterations.
(XLSX)

S2 Table Corrected p-values (*tmax*-statistic) related to S2 Fig (Sensitivity analysis of the 40Hz intrinsic phase asymmetry). Each row indicates the corrected *p*-values of the non-parametric permutation test for a different randomized selection of 38 trials. For reasons of illustration, we here displayed *p*-values in the post-stimulus onset interval 32-64ms.
(XLSX)
