# Supplementary material for: Intrinsic 40Hz-phase asymmetries predict tACS effects during conscious auditory perception
Source: PLoS One. 2019 Apr 3;14(4):e0213996. doi: 10.1371/journal.pone.0213996 (PMC6447177; doi:10.1371/journal.pone.0213996)
Supplement: S1 Text — (DOCX) [file pone.0213996.s001.docx]

**Supporting Information**

**S1 Text. Electric field modeling**

We here derive the formal expressions used to calculate an optimal stimulation of a given location and orientation inside the head. The electric field induced by inserting a unit current at some location (the stimulation electrode) and extracting it some other location (the reference) is well known to be equal to the electric lead field representing the sensitivity of an EEG sensor at that location (with that reference) to source activities. Hence, calculating electric field distributions during tACS stimulation is equivalent to solving the EEG forward problem, for which an analytic expansion of the electric lead field in spherical harmonics for a three-shell volume conductor of realistic shape was used [1]. Within the volume inside the innermost shell a grid with neighboring grid-points having 5mm distance is defined, and EEG forward solutions are calculated for a fixed set of EEG electrodes.

We denote by *L(i,j,k)* the electric potential at the i.th sensor, for the j.th grid-point, induced by a source of unit magnitude pointing into k.th direction with *k*=1,..,3. The electric field induced by a tACS stimulation of magnitude *α(i)* for the i.th sensor induces, apart from an irrelevant constant, an electric field at the *j.th* grid-point in the *k.th* direction equal to

$E\left( j,k \right)=\sum_{i} L\left( i,j,k \right)\alpha(i)$ (10)

The goal is to optimize the stimulation *α(i)* such that the induced electric field is fixed in a specified direction at a specified grid-point inside the head while simultaneously minimizing the stimulation of all other regions and directions. The latter can be defined in various ways, and we here chose to minimize the (square of the) 2-norm of the electric field leading to minimize the cost function

$H=\sum_{j,k} {E(j,k)}^{2}$ (11)

under the constraint

$\sum_{i} L_{0}\left( i \right)\alpha(i)=1$ (12)

where *L_0_(i)* is the topography of an electric dipole at the specified location with specified orientation. It is possible (but not necessary) to choose the location at one of the grid-points, say the m.th. If the source direction is given by *u(k)* with *k=1,..,3* then

$L_{0}\left( i \right)=\sum_{k} L\left( i,m,k \right)u(k)$ (13)

Minimizing the cost function H under the constraint can be solved analytically and results in

$\vec{\alpha}=\frac{1}{\lambda}K^{-1}\vec{L}_{0}$ (14)

with

$K(p,q)=\sum_{j,k} L\left( p,j,k \right)L(q,j,k)$ (15)

and

$\lambda=\vec{L}_{0}^{T}K^{-1}\vec{L}_{0}$ (16)

The above formulation is only valid if *K* is invertible which is not the case if the reference electrode is included in the lead field tensor *L* or if, e.g., the topographies are referenced to common average reference. In such a case the inverse of *K* can be replaced by its pseudo-inverse. A more general, approach is to regularize *K* and make the replacement

$K\to K+\beta id$ (17)

where *id* is the identity matrix in the N-dimensional sensor space. Replacing the inverse of *K* with its pseudo-inverse corresponds to the limit *β→0*, while choosing a finite value for *β*  leads to less total current inserted to the head compromising on the cost function. In this paper we have chosen a rather small regularization of

$\beta={10}^{-4}\frac{tr(K)}{N}$ (18)

**References**

1. Nolte G, Dassios G. Analytic expansion of the EEG lead field for realistic volume conductors. Phys Med Biol. 2005;50: 3807–3823. doi:10.1088/0031-9155/50/16/010
