# Supplementary material for: Intrinsic 40Hz-phase asymmetries predict tACS effects during conscious auditory perception
Source: PLoS One. 2019 Apr 3;14(4):e0213996. doi: 10.1371/journal.pone.0213996 (PMC6447177; doi:10.1371/journal.pone.0213996)
Supplement: S2 Text — (DOCX) [file pone.0213996.s002.docx]

**Supporting Information**

**S2 Text. Supplementary control analyses**

***Atypical laterality indexes during sham-tACS***

Since the structural model of Kimura suggests that participants with a negative laterality index (LI) might exhibit an atypical organization of speech perception and thus an altered interhemispheric communication between auditory cortices [1], we carried out a supplementary analysis to assess if the main results (Fig 4A-B) are robust against potential confounding influences of participants with a negative LI. Therefore, we excluded three participants who scored a negative LI during sham-tACS (LI = -11.8; -2.5; -0.9) and repeated the non-parametric permutation test on the averaged time courses of the intrinsic phase asymmetries (*n*=23). Importantly, the exclusion of these participants again yielded a significant difference between the perceptual outcomes of the phase asymmetries in the post-stimulus onset interval from 44-60ms (*tmax*-method, see S1 Table; Figure A in S1 Figure). Furthermore, the significant circular-linear correlation between tACS-related behavioral modulation and the intrinsic phase asymmetry (*rho*=.5932, *p*=0.0175) during left ear percept in the sham session was not affected by the exclusion of these participants (Figure B in S1 Figure).

***Sample size bias***

The DL paradigm is characterized by a clear behavioral asymmetry as healthy right-handed participants report significantly more syllables perceived through the right ear, which complicates the statistical comparison of left and right ear trials in the form of a sample size bias. Since the statistical comparison of conditions in the EEG source space is additionally hampered as spatial filters, which are applied during LORETA, are biased by unmatched trial numbers [2], we decided to balance the trial numbers across subjects and conditions by randomly subsampling 38 trials out of each subject’s datapool (see Methods, EEG data preprocessing). To confirm that our results were not confined to one specific trial selection, we repeated the trial subsampling procedure and the ensuing non-parametric permutation test 10 times as depicted in the Methods section. Throughout all repetitions, we observed increased intrinsic phase asymmetry during left ear percept compared to right ear percept in a similar time window (40-48ms post-stimulus onset; S2 Figure; *p*-values are displayed in S2 Table).

**References**

1. Kimura D. Functional Asymmetry of the Brain in Dichotic Listening. Cortex. 1967;3: 163–178. doi:10.1016/S0010-9452(67)80010-8

2. Helfrich RF, Knepper H, Nolte G, Sengelmann M, König P, Schneider TR, et al. Spectral fingerprints of large-scale cortical dynamics during ambiguous motion perception. Hum Brain Mapp. 2016;37: 4099–4111. doi:10.1002/hbm.23298
