# Supplementary material for: Intrinsic 40Hz-phase asymmetries predict tACS effects during conscious auditory perception
Source: PLoS One. 2019 Apr 3;14(4):e0213996. doi: 10.1371/journal.pone.0213996 (PMC6447177; doi:10.1371/journal.pone.0213996)
Supplement: S3 Text — (DOCX) [file pone.0213996.s003.docx]

**Supporting Information**

**S3 Text. Test-retest reliability of the laterality index and the intrinsic phase asymmetry at 40Hz**

The circular-linear correlation analysis (Fig 4B) was performed on metrics (laterality index modulation, intrinsic phase asymmetry during sham-tACS) that were assessed on two different days. While previous studies revealed both low [1,2] as well as high [3–5] test-retest reliability values of the right ear advantage (REA) for verbal material during dichotic listening, the test-retest reliability of the intrinsic phase asymmetry at 40Hz has not yet been assessed.

To further discuss the test-retest reliabilities of the primary variables in this work (laterality index, intrinsic phase asymmetry), we took the opportunity to reanalyze unpublished pilot data where 18 right-handed healthy participants (9 female, mean age: 25.77 ± 3.95 years) performed the same dichotic listening task (240 syllables with CV-combinations) on two different days within two weeks during EEG-recording. The EEG recordings were obtained from 64 Ag/Cl electrodes (no amplitude clipping, impedances <10kΩ), referenced to the nose tip) in an equidistant array using slightly abrasive electrolyte gel (Abralyt 2000, Easycap). EEG was sampled at 5000Hz using BrainAmp amplifiers (Brain Products GmbH), amplified in the range of ±16.384 mV at a resolution of 0.5 µV and stored for offline analyses. Sensor space preprocessing and source space analysis (eLORETA; http://www.uzh.ch/keyinst/loreta.htm) were kept identical to the analysis depicted in the Methods section, with a minor exception in that the pilot data (*n*=18) were resampled to 1000Hz instead of 250Hz.

Importantly, the assessment of reliability scores according to classical test theory demands that an equal number of observations is obtained throughout all individuals [6]. Consequently, we kept the randomizing trial subsampling procedure identical to the main experiment to prevent a sample size bias and calculated circular means across the same post-stimulus onset interval (36-56ms) over 35 trials (lowest number across both sessions and subjects) in the *left ear* condition. We hence computed the test-retest reliability by means of a circular-circular correlation (function: *circ_corrcc.m,* CircStat-toolbox [7]). Importantly, the asymmetry values exhibited a high test-retest reliability during left ear percept at 40Hz (*rho*=0.8529, *p*=.0047; Figure B in S3 Figure).

Furthermore, we assessed each participant’s behavioral performance during both sessions (T1 and T2) by means of the laterality index and computed the test-retest reliability using the Matlab-function *cronbach.m (*written and provided by Alexandros Leontitsis, in accordance with [8])*.* Interestingly, this analysis revealed an excellent test-retest reliability of α=.9444 (Figure A in S3 Figure), which is in line with the concept that the LI reflects individual trait differences in hemispheric integration via posterior parts of the corpus callosum.

**References**

1. Pizzamiglio L, De Pascalis C, Vignati A. Stability of Dichotic Listening Test. Cortex. 1974;10: 203–205. doi:10.1016/S0010-9452(74)80010-9

2. Speaks C, Niccum N. Variability of the ear advantage in dichotic listening. J Am Audiol Soc. 1977;3: 52–57.

3. Bakker DJ, Van der Vlugt H, Claushuis M. The reliability of dichotic ear asymmetry in normal children. Neuropsychologia. 1978;16: 753–757.

4. Kraft RH. Laterality and school achievement: interactions between familial handedness and assessed laterality. Percept Mot Skills. 1985;61: 1147–1156. doi:10.2466/pms.1985.61.3f.1147

5. Mahdavi ME, Pourbakht A, Parand A, Jalaie S. Test-Retest Reliability and Minimal Detectable Change of Randomized Dichotic Digits in Learning-Disabled Children: Implications for Dichotic Listening Training. J Am Acad Audiol. 2018;29: 223–232. doi:10.3766/jaaa.16134

6. Clayson PE, Miller GA. Psychometric considerations in the measurement of event-related brain potentials: Guidelines for measurement and reporting. Int J Psychophysiol. 2017;111: 57–67. doi:10.1016/j.ijpsycho.2016.09.005

7. Berens P. CircStat: A MATLAB Toolbox for Circular Statistics. Journal of Statistical Software. 2009;31: 1–21. doi:10.18637/jss.v031.i10

8. Cronbach LJ. Coefficient alpha and the internal structure of tests. Psychometrika. 1951;16: 297–334. doi:10.1007/BF02310555
