# Supplementary figures and images for: Intrinsic 40Hz-phase asymmetries predict tACS effects during conscious auditory perception
Source: PLoS One. 2019 Apr 3;14(4):e0213996. doi: 10.1371/journal.pone.0213996 (PMC6447177; doi:10.1371/journal.pone.0213996)

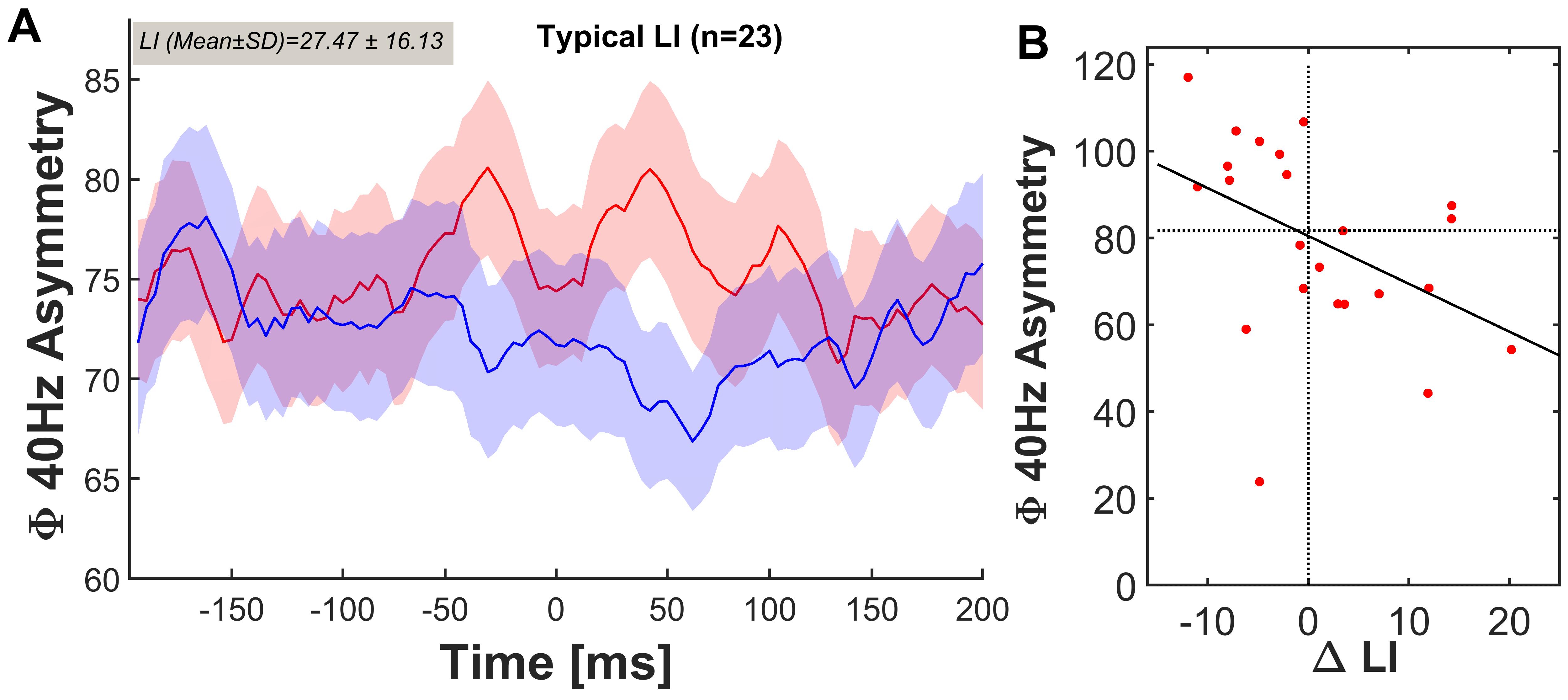

Supplement: S1 Fig — (JPG) [file pone.0213996.s004.jpg]

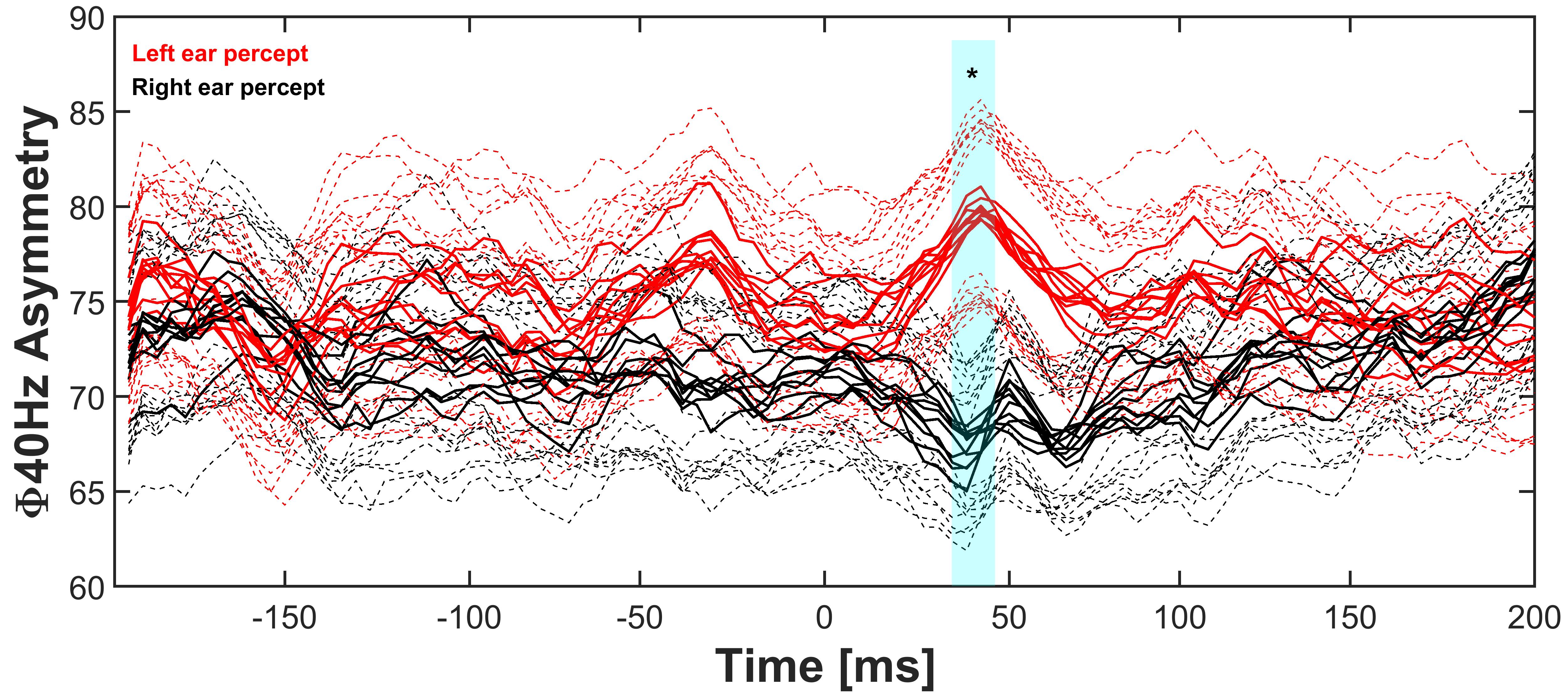

Supplement: S2 Fig — Average time courses (solid lines) of the intrinsic phase asymmetry (related to Fig 4A in the manuscript) with standard errors of the circular means (dashed lines) for 10 different randomized trial selections (M ± SE over 38 trials during left (red lines) and right (black lines) ear percept, respectively). The turquoise-shaded bar highlights that the effect of increased phase asymmetry during left ear percept was present in the marked post-stimulus onset interval throughout all repetitions (maxstat-method; corrected p-values are displayed in S2 Table). (JPG) [file pone.0213996.s005.jpg]

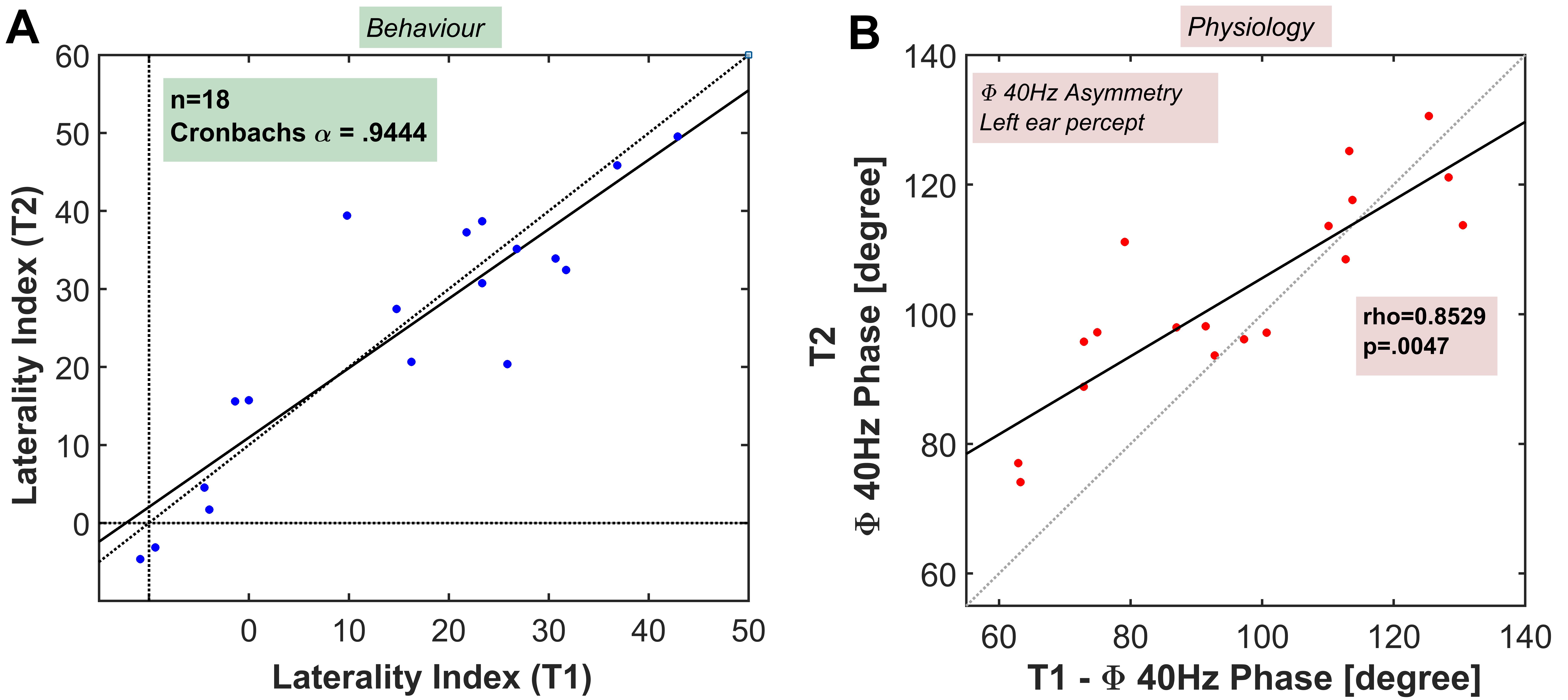

Supplement: S3 Fig — (JPG) [file pone.0213996.s006.jpg]
